# SMRT Sequencing of the Full-Length Transcriptome of the *Coelomactra antiquata*

**DOI:** 10.3389/fgene.2021.741243

**Published:** 2021-10-14

**Authors:** Aiping Deng, Jinpeng Li, Zebin Yao, Gyamfua Afriyie, Ziyang Chen, Yusong Guo, Jie Luo, Zhongduo Wang

**Affiliations:** ^1^ College of Fisheries, Guangdong Ocean University, Zhanjiang, China; ^2^ Guangdong Provincial Key Laboratory of Aquaculture in South China Sea for Aquatic Economic Animal of Guangdong Higher Education Institutes, Fisheries College, Guangdong Ocean University, Zhanjiang, China

**Keywords:** *Coelomactra antiquata*, full-length transcriptome, SMRT sequencing, RNA-seq, function annotation

## Abstract

*Coelomactra antiquata* is an important aquatic economic shellfish with high medicinal value. However, because *C. antiquata* has no reference genome, a lot of molecular biology research cannot be carried out, so the analysis of its transcripts is an important step to study the regulatory genes of various substances in *C. antiquata*. In the present study, we conducted the first full-length transcriptome analysis of *C. antiquata* by using PacBio single-molecule real-time (SMRT) sequencing technology. The results identified a total of 39,209 unigenes with an average length of 2,732 bp, 23,338 CDSs, 251 AS events, 9,881 lncRNAs, 20,106 SSRs, and 2,316 TFs. Subsequently, 59.22% (23,220) of the unigenes were successfully annotated, of which 23,164, 18,711, 15,840, 13,534, and 13,474 unigenes could be annotated using NR, Swiss-prot, KOG, GO, and KEGG databases, respectively. This study lays the foundation for the follow-up research of molecular biology and provides a reference for studying the more medicinal value of *C. antiquata*.

## Introduction

The *Coelomactra antiquata* is a bivalve marine creature that lives in the bottom sand (Kong and Li, 2009). As a wide temperature shellfish, it is predominantly distributed in the western Pacific Ocean, the Indian Peninsula, Japan, and the coast of China. In China, *C. antiquata* is distributed from Liaoning province in the north and Guangxi Zhuang autonomous region in the south (Kong et al., 2007). The meat of the *C. antiquata* is tender, delicious, and nutritious, making it a remarkable species with high economic value (Liu et al., 2006). However, due to excessive fishing in recent decades, the natural population of *C. antiquata* has gradually decreased (He et al., 2021). Fortunately, the artificial breeding of *C. antiquata* has gradually matured in recent years of continuous attempts (Liu et al., 2012; Chen., 2018a; Chen., 2018b).

In addition to research on mitochondrial genomes (Meng et al., 2012, 2013; Shen et al., 2016), the content of previous research mainly focused on its morphological research (Kong et al., 2007), population genetic comparison (Kong and Li, 2007), and organizational composition research of *C. antiquata* (Wu et al., 2019). As well, there was some research on the possible role of this bivalve in disease treatment. For example, treating diabetic mice with different doses of *C. antiquata* extract can reduce the blood glucose concentration of diabetic mice and increase the antioxidant activity of serum (Wen et al., 2015). Also, using a dose of 30 mg/kg of *C. antiquata* polysaccharides on human carcinoma of esophagus cells transplanted in nude mice, its inhibitory rate of 28.85% was recorded (Yang et al., 2015).

According to the above description, there have been many reports on the genetic, morphological, and disease treatment effects of *C. antiquata*, but there are few studies on the transcriptome level. Transcriptome sequencing (RNA-seq) is a technology that uses high-throughput sequencing technology to sequence and analyze all or part of mRNA, small RNA, and no-codingRNA in cells or tissues. RNA-seq can identify genes involved in a variety of biological processes and obtain relevant transcripts in biological processes (De Klerk et al., 2014; Chen et al., 2020). With the continuous development of nucleic acid sequencing technology and the advent of third-generation sequencing technology, the full-length transcriptome can be obtained more simply and accurately (Schadt et al., 2010). Compared with the first-generation and second-generation sequencing technologies, the third-generation sequencing technology can directly obtain the full-length transcript sequence without assembling, which can truly reflect the transcriptome information of the sequenced species (Li et al., 2008; Bleidorn, 2016; Jia et al., 2020). This study used PacBio’s single-molecule real-time (SMRT) sequencing technology to generate comprehensive full-length transcriptome of *C. antiquata*. We then systematically carried out structural analysis and functional annotation of those full-length transcriptomes to obtain a large amount of usable sequence information. From this sequence information, we can see that many transcripts of *C. antiquata* have signal transduction, synthesis, and metabolism functions, indicating that there may be many biologically active substances in *C. antiquata* that participate in the life processes. This study will provide data for follow-up study of certain functional genes, molecular biology research, and exploration of possible biomedical functions of *C. antiquata*.

## Materials and Methods

### Sample Collection for Iso-Seq

One *C. antiquata* (Shell length: 87 mm, shell width: 65 mm, shell height: 40.5 g) sampled from Leizhou in Guangdong Province. Tissues including blood, mantle, adductor muscle, lip, foot, gill, inlet pipe, outlet pipe, kidney, intestine, liver, and gonad were rapidly collected, immediately frozen in liquid nitrogen, and then stored at −80°C for preservation until RNA extraction.

### RNA Extraction

Total RNA was separately extracted from these tissue samples (Jia et al., 2020; Zheng et al., 2020). The purity, concentration, and absorption peak of the extracted RNA were measured using a NanoDrop 2,000 spectrophotometer (Thermo Fisher Scientific Inc., United States). Agarose gel electrophoresis was mainly used to detect the genomic contamination, purity of samples, and the Agilent 2,100 was used to determine the RIN value accurately detecting the integrity of RNA. When the test results met the requirements, RNA samples from 12 tissue were mixed together for the following library preparation.

### Library Preparation and SMRT Sequencing

The Clonetech SMARTerTM PCR cDNA Synthesis Kit was used to reverse transcribe the pooled total RNA into cDNA. Afterwards, polymerase chain reaction (PCR) was employed to amplify the cDNA and using primers with Oligo dT. The amplified cDNA was purified with PB magnetic beads. After purification, all full-length cDNAs were end-repaired and connected with SMRT dumbbell adaptors. Exonuclease digestion was implemented to remove sequences that failed to ligate to the adapters. The resulting sequences were purified again. Finally, a SMRTbell library was constructed. Prior to sequencing, the accurate quantification of the libraries was assessed by Qubit 3.0 and the size of the libraries were detected by Agilent 2,100. Then the full-length transcriptome was sequenced with PacBio sequencer.

### Sequencing Data Processing

The raw sequencing data were processed using the SMRTlink (Hon et al., 2020) software with the parameters: --min_passes 3; --min_length 50; --max_length 15,000. The high-quality sequencing reads produced by a single molecule in the sequencing process are called polymerase read, and the polymerase reads remove the sequencing adapters to form subreads. A circular consensus sequence (CCS) was obtained from the subreads. The CCS sequence was checked to see whether it contained 5′primer, 3′primer, and polyA. Their positional relationships were assessed and later divided the CCS sequence into three categories: the full-length sequence (FL), the full-length non-concatemer sequence (FLNC), and the full-length non-chimeric sequence with polyA. ICE of SMRTlink software was used to cluster FLNC sequences and obtain a set of cluster consensus sequences. Further the sequences were polished by Arrow algorithm (Cao et al., 2020) and obtained the FLNC polished high quality consensus Sequences. Finally, CD-HIT (Li & Godzik, 2006) software (parameters: -c 0.99; -G 0; -aL 0.90; -AL 100; -aS 0.99; -AS 30) was used to perform clustering and de-redundancy. The unigenes from high quality full-length transcripts were used for subsequent analysis.

### Function Annotation

To obtain basic annotations information, non-redundant transcripts were annotated against six different databases, namely, Non-supervised Orthologous Groups (NR), EuKaryotic Orthologous Groups (KOG), Gene Ontology (GO), Kyoto Encyclopedia of Genes and Genomes (KEGG), Swiss-Prot, and Pfam databases. DIAMOND (Buchfink et al., 2014) software (parameters: --more-sensitive; -k 10; -e 1e-5) was used for NR, Swiss-Prot, KOG, GO, KEGG databases analyses, and the Hmmer package (Nguyen et al., 2016) with default parameters utilized for Pfam database analyses.

### Structure Analysis

#### CDS, LncRNA, and TFs Prediction

TransDecoder software (Haas et al., 2013) (parameters: -G universal; -S; -m 100) was used to predict the coding sequences (CDS) of transcripts. Transcripts longer than 200 nucleotides (nts) were used for the long noncoding RNA (lncRNA) prediction. Four methods, Coding Potential Calculator 2 (CPC2) (Kang et al., 2017) (default parameters), Coding-Non-Coding Index (CNCI) (Sun et al., 2013) (parameters: -m pl), Coding Potential Assessment Tool (CPAT) (Wang et al., 2013) (default parameters), and PLEK (Li et al., 2014) (parameters: -minlength 200), were integrated to identify lncRNA in the transcripts and depict the intersection of the results predicted by the four methods. For the TF (transcription factor) analysis, we used DIAMOND (Buchfink et al., 2014) software to align the sequences to the AnimalTFDB (animal transcription factor database) for TFs prediction.

#### Simple Sequence Repeat Analysis

With the default parameters of MISA 1.0 (Beier et al., 2017), all SSRs present within the transcriptome sequences were identified and count the regional distribution of some SSRs. In the process of identification, the minimum value of repeat number varied with different repeat units per unit sizes and their minimum number of repetitions were: 1–10, 2–6, 3–5, 4–5, 5-5, and 6–5. For instance, 1–10 indicates that a single nucleotide must be repeated at least 10 times to be detected. The SSR were divided into seven types: Mono-, Di-, Tri-, Tetra-, Penta-, Hexa-, and compound SSR.

#### Alternative Splice Prediction

In this study, IsoSeq_AS_de_novo software (Palareti et al., 2016) with default parameters was used to perform Alternative splice analysis of the non-redundant sequences, and this software used a method that does not require reference sequences to detect AS isoforms.

## Result

### SMRT Sequencing Data Analysis

**FIGURE 1:**
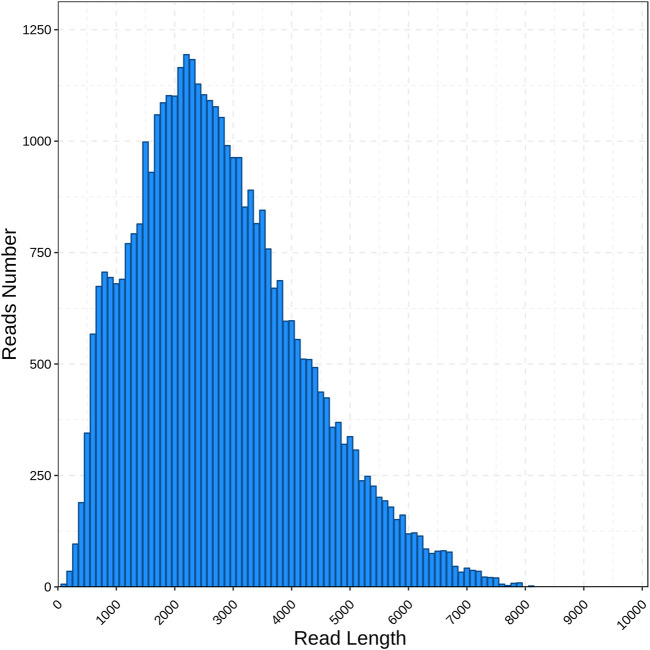
Length distribution of unigenes obtained from *C. antiquata* SMRT library.

By using the PacBio Sequel II sequencing platform, we obtained 956,679 of polymerase read (about 112.26 Gb). In a total of 87,338,730 subreads, an average length of the subread was 1,173 bp and an N50 length of 2,347 bp. After self-correction among subreads, 660,201 CCS reads were gained in which a mean of the CCS read length was 2,416 bp. The amount of CCSs for each transcript ranges from 2 to 24,103, with an average of 9, and the average accuracy of the obtained CCS data was 0.99951. By detecting the sequences, 5,057,806 CCSs were identified as full-length reads and 495,198 were identified as FLNC reads with an average length was 2,423 bp and an N50 length of 3,057 bp. Then, 41,056 polished transcripts were obtained. Finally, the redundant reads were removed by CD-HIT and 39,209 unigenes with a mean length of 2,732 bp were obtained (Table 1). The length distribution of unigenes is as shown in Figure 1. The majority of unigenes were between 1,000 and 4,000 bp (28,643, 73.05%), and the longest unigenes was about 8,000.

**TABLE 1:** Description of full-length sequencing in *C. antiquata*.

Type	Total number	Min length	Average length	Max length	N50
Polymerase read	956,679	51	117,339	425,391	189,911
Subread	87,338,730	51	1,173	274,252	2,347
CCS	660,201	48	2,416	14,948	3,136
FLNC	495,198	50	2,423	8,687	3,057
Unigenes	39,209	66	2,732	8,074	3,324

### Functional Annotation

**FIGURE 2:**
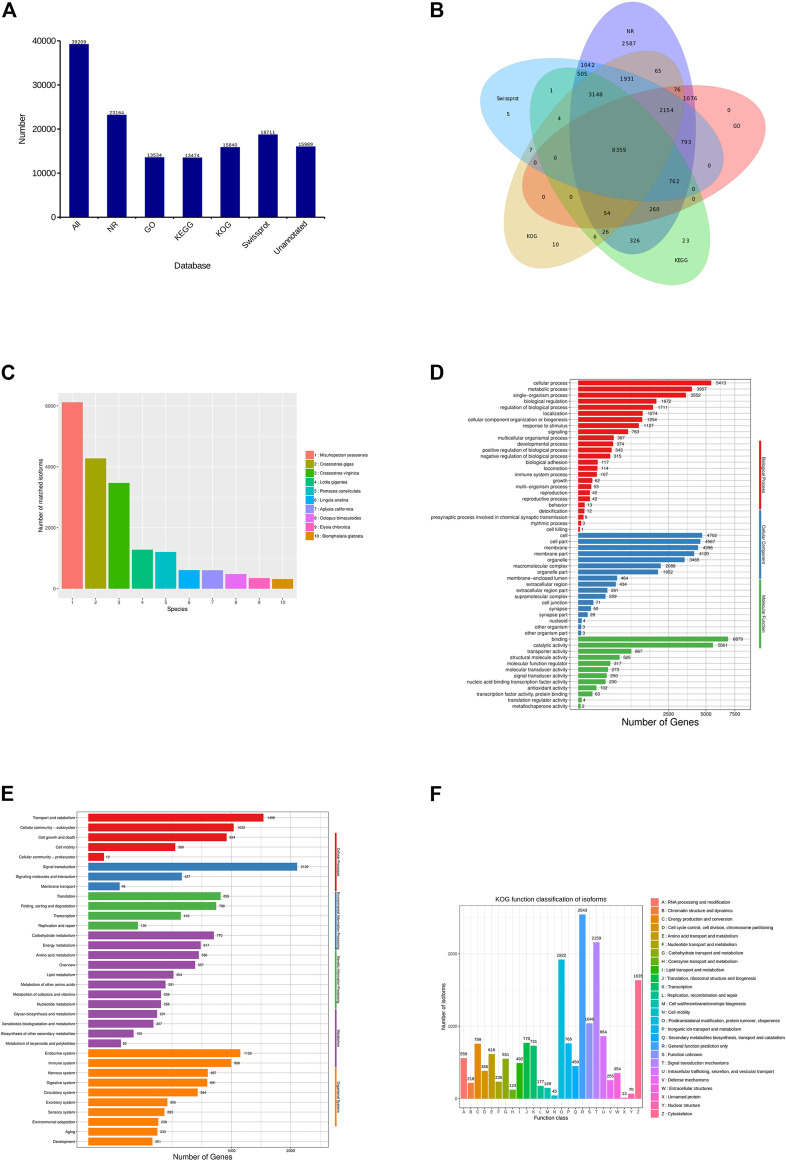
Functional annotation in *C. antiquata.* **(A)** Statistics of the transcripts annotated in different databases. **(B)** Venn diagram of annotations in NR, GO, KEGG, KOG, and SwissProt databases. **(C)** Distribution of the top 10 species with matched transcripts in the NR database. **(D)** Distribution of GO terms for all annotated transcripts in biological process, cellular component, and molecular function. **(E)** KEGG pathways enriched by transcripts. **(F)** COG categories of the transcripts.

In all, 39,208 transcripts were annotated in the five databases, of which 23,264 (59.33%), 13,534 (34.52%), 13,474 (34.36%), 15,840 (40.40%), and 18,711 (47.72%) transcripts were, respectively, matched to the NR, GO, KEGG, KOG, and Swiss-Prot databases (Figure 2A). A total of 8,359 (22.84%) transcripts were annotated in all the databases (Figure 2B). Aligning each transcript with the homologous sequence of the NR library, it was determined which species the sequence with the best comparison result belongs to, and count the number of homologous sequences aligned with each species. According to statistics, the species with the most homology was *Mizuhopecten yessoensis* (6,118 transcripts), followed by *Crassostrea gigas* (4,276), *Crassostrea virginica* (3,471), and *Lottia gigantea* (1,281) (Figure 2C).

According to GO classification statistics of the transcripts, the annotated results included three broad categories: Biological process (22,917 transcripts), Cellular component (26,837) and Molecular function (15,073). The Cellular process (5,413, 39.99%), Cell (4,702, 34.74%), and Binding (6,879, 50.82%) were the most annotated transcripts in the three categories mentioned above (Figure 2D).

In KEGG pathways, the transcripts were assigned to five main categories: Cellular processes (3,845 transcripts), Environmental information processing (2,604), Genetic information processing (2,192), Metabolism (4,291), and Organismal systems (5,359). Signal transduction (2,129, 15.80%) was the largest group of transcripts, followed by Transport and catabolism (1,498, 11.12%) (Figure 2E).

The KOG classifications of the transcripts obtained clusters of 26 functional categories (Figure 2F). A total of 2,543 (16.05%) transcripts were annotated in General function prediction only, which is the most among functional categories. Next was the Signal transduction mechanisms (2,159, 13.63%).

### Structure Analysis

#### CDS Prediction

**FIGURE 3:**
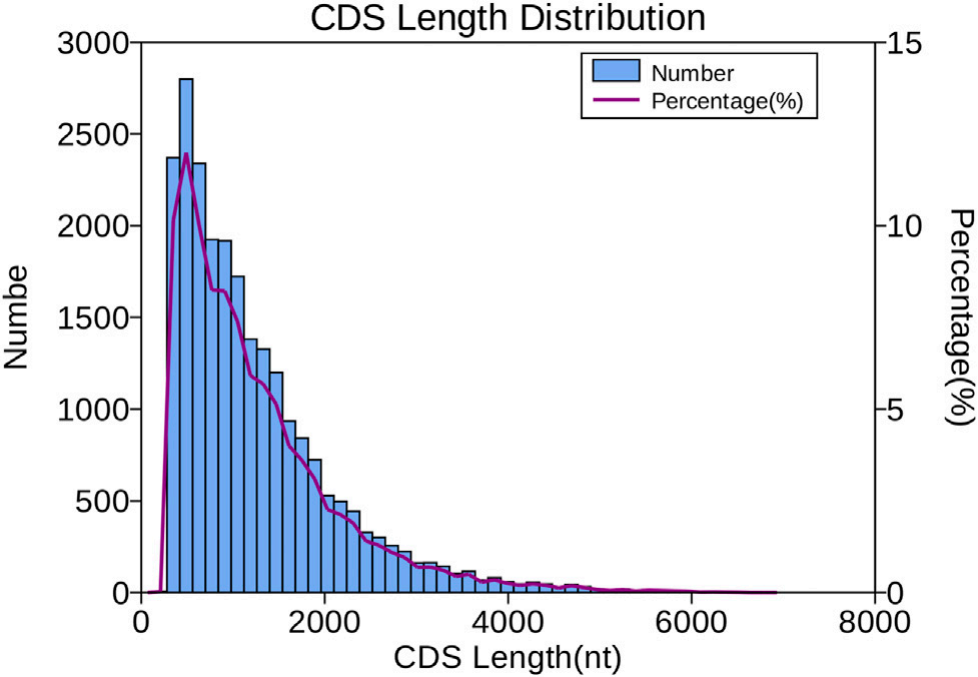
Length distribution of CDSs.

The number and length of 5′UTR, 3′UTR, and CDS were identified by transdecoder software. In total, 15,555 transcripts were predicted in the 5′UTR, 20,550 in the 3′UTR, and 23,338 in the CDS. As shown in Figure 3, most of the CDS, 19,643 transcripts (84.16%) lengths, were less than 2,000 nt, 14.13% ranged from 2,000 to 4,000 nt (3,299 transcripts), and only 396 transcripts representing 1.69% were over 4,000 nt.

#### Identification of lncRNAs

**FIGURE 4:**
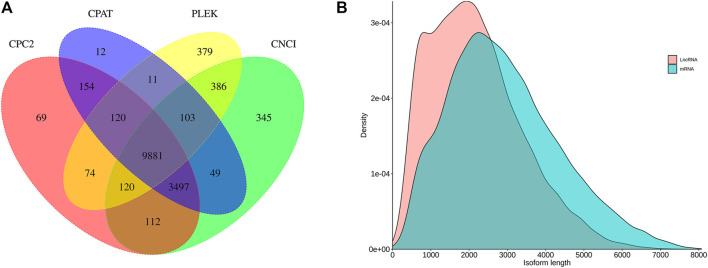
Long non-coding RNA (lncRNA) prediction. **(A)** Venn diagram of lncRNAs predicted by CNCI, CPAT, CPC2, and PLEK methods. **(B)** Length distribution of identified lncRNAs and mRNA in *C. antiquata*.

We identified 14,493, 13,827, 14,027, and 11,074 lncRNAs by CNCI, CPAT, CPC2, and PLEK, respectively. The results of these four methods were integrated and 9,881 lncRNA transcripts were predicted totally (Figure 4A). By comparing the length distribution density of lncRNA and original mRNAs, it was found that there were more lncRNAs with lengths between 1,000 and 2,000 nt than mRNAs, and the longest predicted lncRNA does not exceed 8,000 nt (Figure 4B).

#### SSR Analysis

**FIGURE 5:**
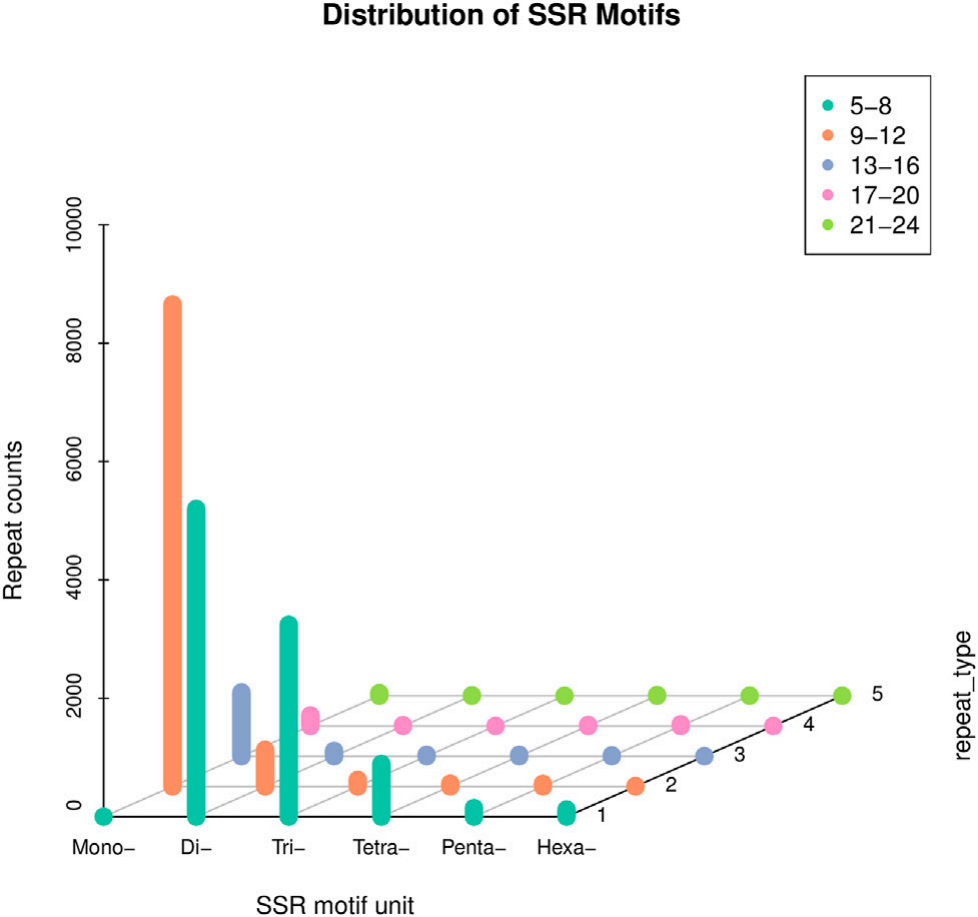
Summary of SSR types in the *C. antiquata* transcriptome.

SSR analysis of the transcriptome revealed a total of 20,106 SSRs using MISA 1.0 software. Upon careful scrutiny of the obtained SSRs, the most predominant was the mono-nucleotide repeats (9,443), which accounted for 46.96%, followed by the di-nucleotide repeats (5,932), representing 29.50%, and tri-nucleotide repeats (3,379), accounted for 16.80%. However, tetra-nucleotide, penta-nucleotide, and hexa-nucleotide repeats accounted for a very small number, 4.92, 1.15, and 0.64%, respectively. Besides, the number of repetitions for most SSRs were 5-8 and 9–12 (Figure 5). Since some transcripts cannot predict the CDS, the total number of SSRs that can be counted in different regions was 9,804. Among the 9,804 SSRs, the number in the 3′UTR was the most (8,024), followed by the CDS (1,354), and the 5′UTR (426) was the least (Table 2).

**TABLE 2:** Regional distribution of some SSRs in the full-length transcriptome of *C. antiquata.*

Type	Number	Ratio (%)	5′UTR	CDS	3′UTR
Mono-	4,179	42.63	172	50	3,957
Di-	2,696	27.50	117	38	2,541
Tri-	2,234	22.79	93	1,245	896
Tetra-	501	5.11	19	4	478
Penta-	116	1.18	24	1	91
Hexa-	78	0.80	1	16	61

#### Transcription Factor Prediction

**FIGURE 6:**
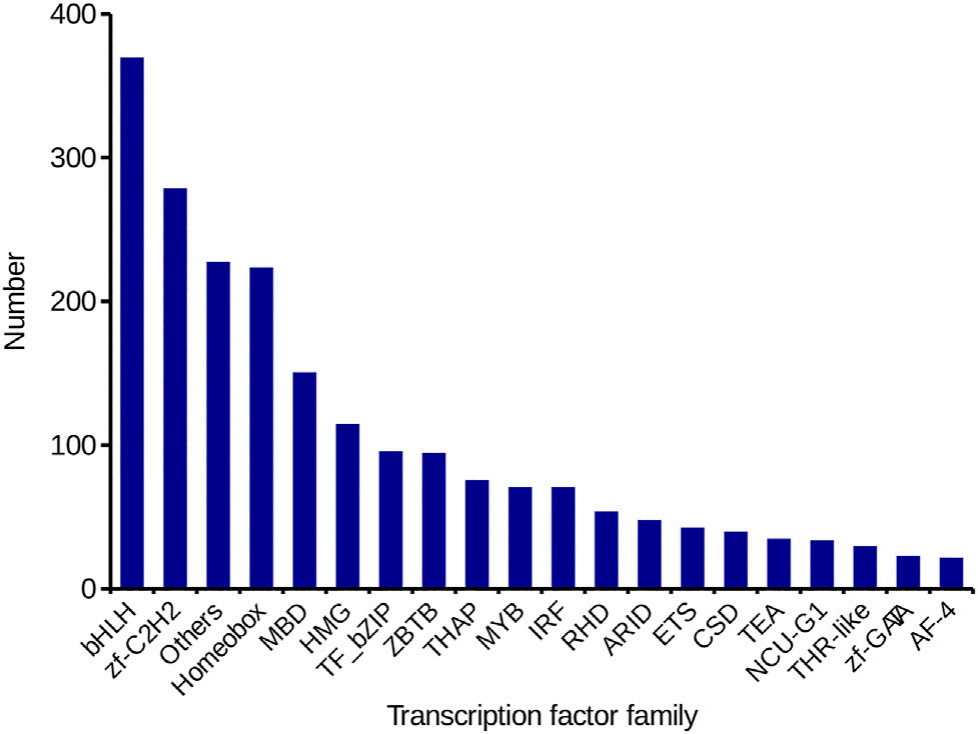
Identification of TFs in*C. antiquata*. The number and family of TFs were predicted by SMRT.

TF is a key factor in regulating gene expression in animals. In this study, 2,316 TFs from 59 TF families were identified by DIAMOND software. List the top 20 TF families in Figure 6, the BHLH family (369, 15.93%) was the most represented, followed by the zf-C2H2 family (278, 12.00%).

### Alternative Splice Prediction

A total of 251 AS events were detected via the IsoSeq_AS_de_novo in all unigenes obtained by SMRT sequencing. Due to the lack of an available *C. antiquata* reference genome, it is necessary to further characterize the types of AS events in future studies.

## Discussion

PacBio RNA-seq has fast sequencing speed, high accuracy, and long readings. Because of the advantages of PacBio RNA-seq, it has been widely used in various species research (Ma et al., 2019; Xue et al., 2019; Luo et al., 2020; Zheng et al., 2020; Chen et al., 2021). Because *C. antiquata* is an important economic shellfish with high medicinal potential, there are many studies now. The present study provides the first full-length transcriptome resource for *C. antiquata* using PacBio single-molecule long-read sequencing technology. By processing and analyzing the sequenced data, a total of 39,209 unigenes were finally identified, with an average length of 2,732 bp. In previous studies, some researchers used the Illumina platform to sequence the transcriptome, and obtained the second-generation transcriptome data of the *C. antiquata* (Yi et al., 2019). The second-generation sequencing identified 214,732 unigenes with a mean length of 616.2 bp. Compared with the results measured by second-generation sequencing, the total number of transcripts obtained by the SMRT sequencing technology in this study is larger and the average length is longer.

In addition, the unigenes obtained were functionally annotated in databases, and 59.22% of the unigenes were successfully annotated. The percentage is not very high, and the possible reason is that there are few studies on molecular biology of this shellfish in the past, the data collected in the database is incomplete, and the genomic information of *C. antiquata* has not yet been referenced. According to the NR annotation situation, *Mizuhopecten yessoensis* has the most homologous sequences annotated, which reflects the high affinity between *C. antiquata* and *Mizuhopecten yessoensis*. It also provides a valuable data basis for the detailed comparison of gene expression between the two species in the future. Among the function statistics against KOG, KEGG, and GO database, the number of transcripts annotated in KOG was the largest. More transcripts are involved in intracellular signal transduction and play a role in the endocrine system to participate in the metabolism of various substances, which proves that there may be many biologically active substances in *C. antiquata* that can be excavated and used in biomedicine.

We also analyzed the structure of the de-redundancy transcripts. A total of 20,106 SSRs and 9,881 lncRNAs were predicted. SSR is widely used in genetic diversity testing, genetic map construction, Gene expression regulation, etc. (Tranbarger et al., 2012; Chen et al., 2014). Compared with the number of SSRs and SSR types obtained by sequencing of the transcriptome of the *Tegillarca granosa*, the number of SSR present in *C. antiquata* was less, and its SSR di-nucleotide repeats were the most (H. Chen et al., 2017). These differences may be related to the different tissue specificities of the two shellfishes. Besides, this study predicted the number of TFs and the detailed family classification of *C. antiquata,* The BHIH family has the largest number. BHLH TFs are the most widespread category in eukaryotes, and they can participate in various processes in cells, such as regulating carbohydrate response genes (Yu et al., 2021), which may indirectly affect the synthesis of various biologically active substances.

In summary, this study successfully constructed a high-quality full-length transcriptome of *C. antiquata*, and preliminarily analyzed its transcriptome structure and functional characteristics, obtained the relevant annotation function of transcripts in the database, and enriched the genetic information of this species. It has laid a solid foundation for the mining and utilization of later functional genes and other molecular biology research.

## Conclusion

We applied PacBio SMRT sequencing platform to obtain a large number of full-length transcriptome data of *C. antiquata* for the first time. The number and mean length of the unigenes from SMRT sequencing were much better than those from Illumina sequencing. And through structural analysis and functional annotation of the obtained full-length transcripts, gene function and gene structure information can be obtained more comprehensively. The acquisition of the full-length transcripts provides molecular biology data for *C. antiquata*, which lacks genomic information. As a species with high medicinal value, in the future, the full-length transcriptome data can be combined with the second-generation sequencing results to conduct further research on the medical effects of its internal substances.

## Data Availability

The datasets provided in this study can be found in the online repository: NCBI SRA database, with the accession number SRR15211944. The repository and accession number can be found at https://www.ncbi.nlm.nih.gov/sra.
